# Supplementary material for: Single-cell transcriptome reveals highly complement activated microglia cells in association with pediatric tuberculous meningitis
Source: Front Immunol. 2024 Apr 30;15:1387808. doi: 10.3389/fimmu.2024.1387808 (PMC11091396; doi:10.3389/fimmu.2024.1387808)
Supplement: Supplementary file 1 [file DataSheet_1.zip › Table 1 .docx]

Supplementary Material

## Supplementary Figures


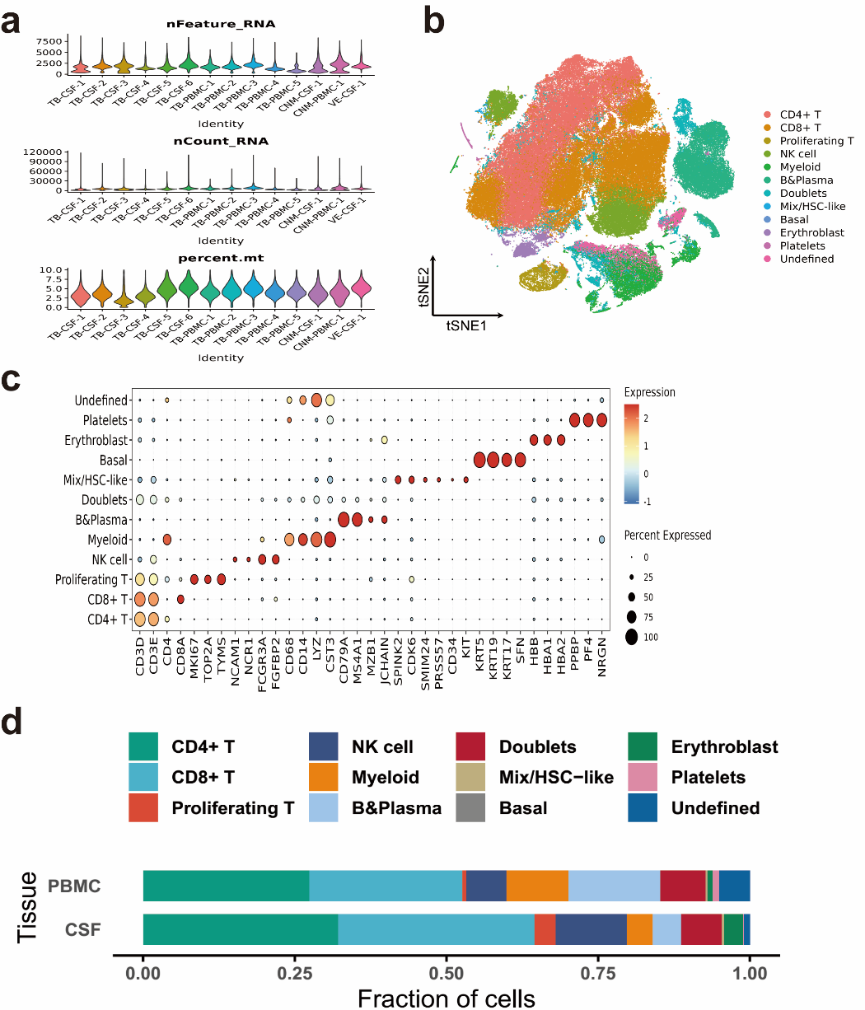


**Supplemental Figure 1 Global profiling of paired PBMCs and CSF cells with TBM.** (a) The number of UMIs (nUMIs, Unique molecular identification) and genes (nGene) identified and the proportion of reads mapping to mitochondrial (mt) genes in all samples (n = 8). (b) Single-cell mapping of tSNE, with each cell color-coded for the relevant cell type. (c) Dot plots depicting selected marker gene expression in cell clusters. The dot size encodes the percentage of cells expressing the gene, while the color encodes the average level of gene expression per cell. (d) The relative proportion of cells for the main cell types comprising PBMCs and the CSF isolated from TBM patients (n = 6).


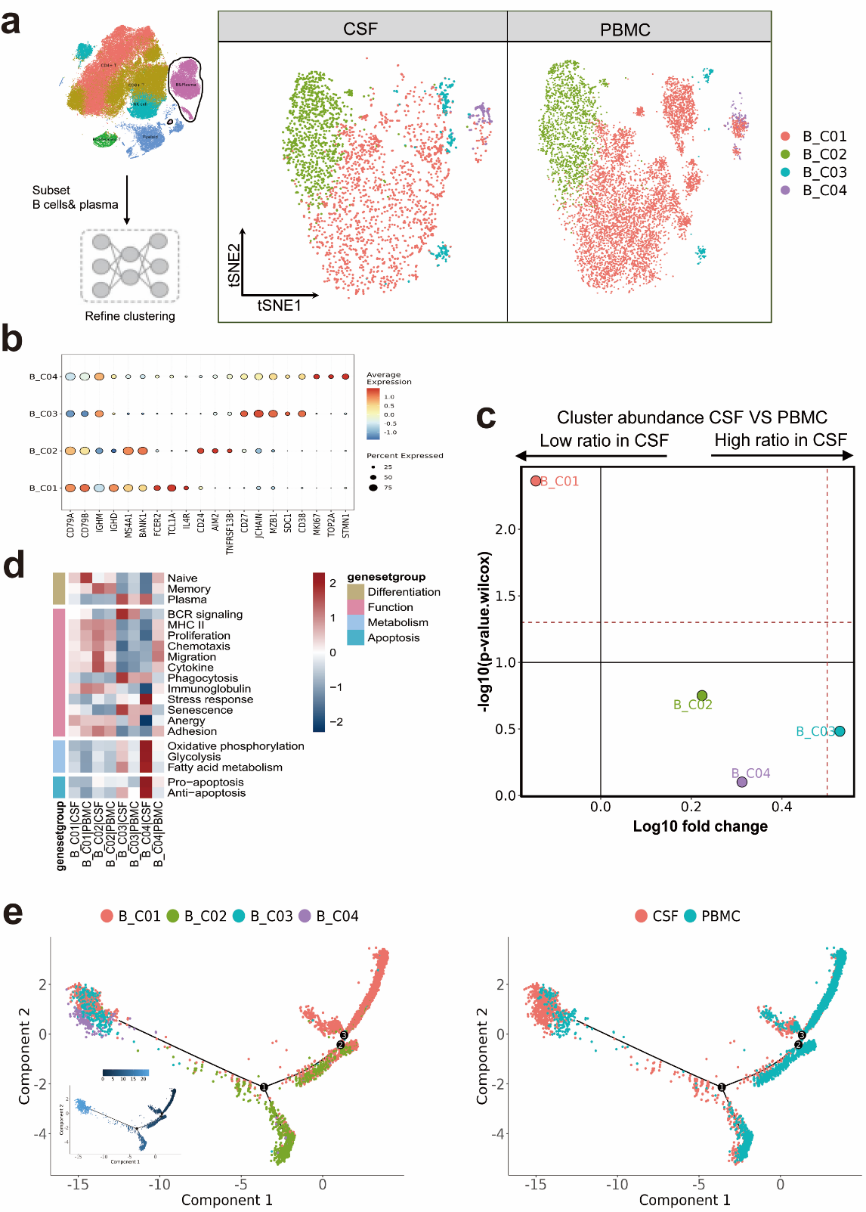


**Supplemental Figure 2 A scRNA-seq identified four B cell subsets in paired PBMCs and CSF.** (a) Subset strategies and flow diagrams of B-cell lineages from paired PBMCs and CSF cell samples. (b) Dot plots showing marker gene expression for each B-cell subcluster. (c) Volcano plots showing differences in CSF versus PBMC B-cell cluster abundance based on t-tests, plotted as the fold change (log10) versus the p-value (- log10). (d) Heatmaps showing the expression of 20 curated gene signatures (see Supplementary Table 8) in B-cell clusters. (e) Monocle2 analysis of B-cell clusters pseudotimes: associated cell types and corresponding states (PBMC, n = 6; CSF, n = 5) are shown.


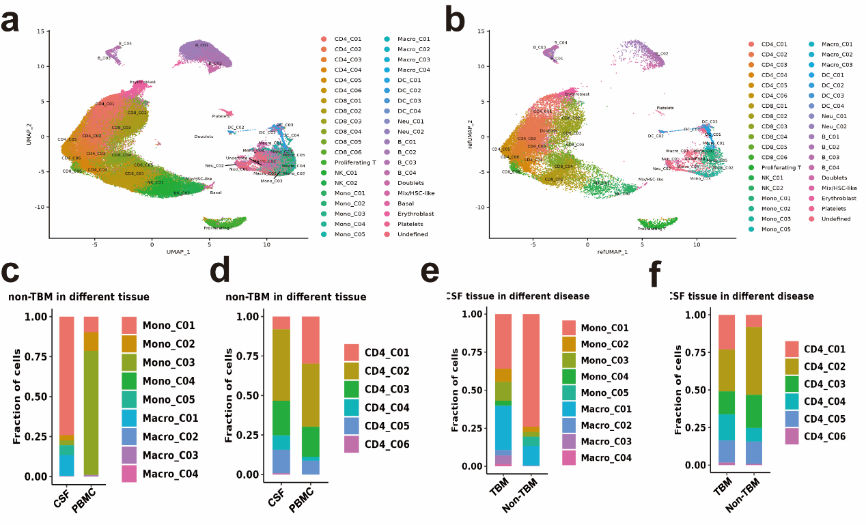


**Supplemental Figure 3 scRNA-seq reveals similarities and differences between TBM and non-TBM in paired PBMCs and CSF.** (a-b) UMAP view of all cell clusters in TBM patients (n=6; a) and Non-TBM patients (n=2; b). (c) Cell proportions of myeloid cell subsets from PBMCs and CSF cells isolated from Non-TBM patients. (d) Cell proportions of CD4 T-cell subsets in PBMCs and CSF cells isolated from Non-TBM patients. (e) Cell proportions of CD4 T-cell subsets in CSF cells isolated from TBM patients (n=6) and Non-TBM patients (n=2). (f) Cell proportions of myeloid cell subsets in CSF cells isolated from TBM patients (n=6) and Non-TBM patients (n=2).

## Supplementary Tables

Supplementary Tables have been uploaded separately on submission.

Supplemental Table 1. TBM patient medication and time, and ELISA validation in this study

Supplemental Table 2. Median UMI and gene counts for all single-cell sequencing samples

Supplemental Table 3. Percentage of each cell for all single-cell sequencing samples

Supplemental Table 4. Differentially expressed genes by cell subsets

Supplemental Table 5. Cell subtype marker list

Supplemental Table 6. Cell subsets enriched for specific markers or marker genes from published datasets

Supplemental Table 7. Comparison of differentially expressed genes between CSF and PBMC cells.

Supplemental Table 8. Gene signatures enriched for cellular subsets of functional modules

Supplemental Table 9. Gene set of the pathway correlation in Macro_C01
